# Third case of gallbladder hemangioma associated with gallstones: a comprehensive literature review

**DOI:** 10.1093/jscr/rjaf049

**Published:** 2025-02-14

**Authors:** Imane Boujguenna, Soumia Moujane, Fatima Boukis, Soufiane Abdouh, Ahmed Elguazzar

**Affiliations:** Guelmim Faculty of Medicine and Pharmacy, Ibn Zohr Agadir University, Guelmim 81000, Morocco; Guelmim Faculty of Medicine and Pharmacy, Ibn Zohr Agadir University, Guelmim 81000, Morocco; Al AMAL Pathological Anatomy Laboratory, Guelmim 81000, Morocco; Private medical practice, Marrakesh 40000, Morocco; General Surgery Department, Moulay Elhassan military hospital. Guelmim 81000, Morocco

**Keywords:** hemangioma, gallbladder, gallstones, histopathology

## Abstract

Gallbladder hemangiomas are extremely rare benign tumors. Eighteen cases have been reported in the literature, with only two associated with gallstones. We report the third case of a gallbladder hemangioma associated with gallstones in a 54-year-old Moroccan woman, along with a comprehensive review of the literature. Gallbladder hemangiomas are likely underdiagnosed, underscoring the need for careful examination of cholecystectomy specimens.

## Introduction

Hemangiomas are benign vascular tumors primarily located in the skin. Gallbladder hemangiomas are exceptionally rare, with only 18 cases reported in the literature, including 2 associated with gallstones [1]. We present the third case of gallbladder hemangioma associated with gallstones in a 54-year-old Moroccan woman.

## Case report

**Figure 1:**
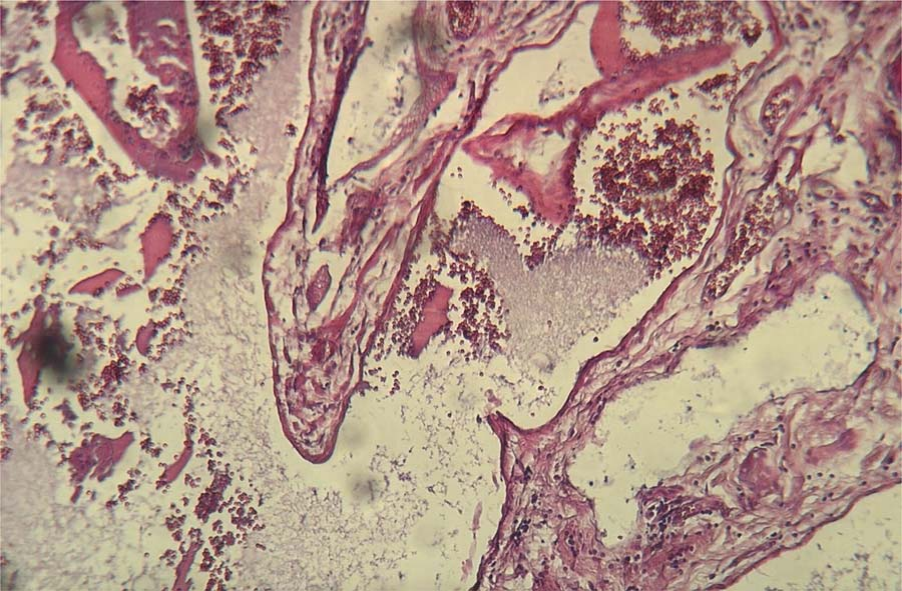
Histopathological examination showing ectatic blood vessels containing red blood cells.

**Figure 2:**
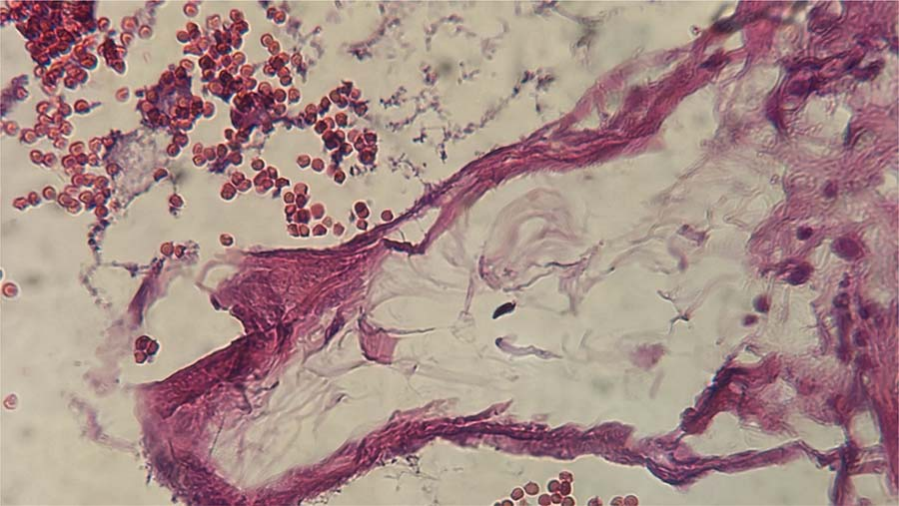
The vessels were lined by a single layer of regular endothelium.

A 54-year-old Moroccan woman with no significant medical history presented with right upper quadrant pain lasting for two months, accompanied by nausea but no fever, weight loss, or other systemic or digestive symptoms. Abdominal ultrasonography revealed gallstones. The patient underwent laparoscopic cholecystectomy. Macroscopic examination revealed a gallbladder measuring 8.6 cm in length and 2.3 cm in width, with a slightly thickened wall and the presence of gallstones. Microscopic examination showed chronic diverticular cholecystitis and a vascular proliferation measuring 10 mm, composed of variably sized, often ectatic blood vessels containing red blood cells (Fig. 1). The vessels were lined by a single layer of regular endothelium (Fig. 2). The patient’s postoperative course was uneventful.

## Discussion

Hemangiomas are predominantly located in the skin, and gallbladder hemangiomas are exceedingly rare. We identified 18 cases in the literature, 2 of which were associated with gallstones (Tables 1 and 2), with our case being the third. Clinically, gallbladder hemangiomas are usually asymptomatic but can present with abdominal pain [1, 2], hemoperitoneum [17], or jaundice [18]. Abdominal ultrasonography, the initial imaging modality for hepatobiliary diseases, may fail to detect hemangiomas, particularly when gallstones are present, as seen in our case. Gallstones can explain the symptoms or pose a differential diagnostic challenge with neoplastic pathologies [18]. Macroscopically, gallbladder hemangiomas can be asymptomatic or appear as violaceous hemorrhagic tumors [1]. Microscopically, they resemble hemangiomas in other locations, showing vascular proliferation with ectatic vessels containing red blood cells and lined by regular endothelium. The histopathological diagnosis is generally straightforward, but in cases of endothelial detachment, immunohistochemistry using endothelial markers can aid diagnosis. The treatment is surgical, consisting of cholecystectomy. In cases with bleeding risk or suspected malignancy, laparotomy exploration is recommended [18]. Our case represents the third reported instance of gallbladder hemangioma associated with gallstones. Gallbladder hemangioma is an extremely rare benign tumor that is likely underdiagnosed. Careful macroscopic and microscopic examination of cholecystectomy specimens is essential for accurate diagnosis.

**Table 1:** Gallbladder hemangioma coexisting with gallstones

Case	Year	Country	Age	Gender
[1]	2022	Italy	76	M
[2]	2022	India	45	F
Our case	2024	Morocco	54	F

**Table 2:** Gallbladder hemangioma

Case	Year	Country	Age	Gender
[3]	1969			
[4]	1973			
[5]	1987		11	F
[6]	1997			
[7]	1997	Spain	50	F
[8]	2005	Italy	49	F
[9]	2016		51	F
[10]	2017	China	44	M
[11]	2018	USA		
[12]	2018	France		
[13]	2018	USA		
[14]	2019	USA	80	F
[15]	2019	Korea	53	M
[16]	2019	Japan	75	M
[17]	2021	India	62	M
[18]	2024	Turkey	49	F
